# Vitamin D levels and perinatal depressive symptoms in women at risk: a secondary analysis of the mothers, omega-3, and mental health study

**DOI:** 10.1186/s12884-016-0988-7

**Published:** 2016-08-03

**Authors:** Jennifer Anne Williams, Vivian C. Romero, Chelsea M. Clinton, Delia M. Vazquez, Sheila M. Marcus, Julie L. Chilimigras, Susan E. Hamilton, Lucy J. Allbaugh, Anjel M. Vahratian, Ronald M. Schrader, Ellen L. Mozurkewich

**Affiliations:** 1 Department of Obstetrics and Gynecology, St. Joseph Mercy Health System, Ypsilanti, MI USA; 2 Spectrum Health Maternal Fetal Medicine, Grand Rapids, MI USA; 3 Department of Obstetrics and Gynecology, Michigan State University College of Human Medicine, East Lansing, MI USA; 4 Department of Obstetrics and Gynecology, University of Michigan, Ann Arbor, MI USA; 5 Department of Pediatrics, University of Michigan, Ann Arbor, MI USA; 6 Department of Psychiatry, University of Michigan, Ann Arbor, MI USA; 7 Clinical and Translational Science Center, University of New Mexico Health Sciences Center, Albuquerque, NM USA; 8 Department of Obstetrics and Gynecology, University of New Mexico Health Sciences Center, University of New Mexico, Albuquerque, NM 87131 USA

**Keywords:** Depression, Pregnancy, Vitamin D

## Abstract

**Background:**

Vitamin D insufficiency may be associated with depressive symptoms in non-pregnant adults. We performed this study to evaluate whether low maternal vitamin D levels are associated with depressive symptoms in pregnancy.

**Methods:**

This study was a secondary analysis of a randomized trial designed to assess whether prenatal omega-3 fatty acid supplementation would prevent depressive symptoms. Pregnant women from Michigan who were at risk for depression based on Edinburgh Postnatal Depression Scale Score or history of depression were enrolled. Participants completed the Beck Depression Inventory (BDI) and Mini International Neuropsychiatric Interview at 12–20 weeks, 26–28 weeks, 34–36 weeks, and 6–8 weeks postpartum. Vitamin D levels were measured at 12–20 weeks (*N* = 117) and 34–36 weeks (*N* = 112). Complete datasets were available on 105 subjects. Using regression analyses, we evaluated the relationship between vitamin D levels with BDI scores as well as with MINI diagnoses of major depressive disorder and generalized anxiety disorder. Our primary outcome measure was the association of maternal vitamin D levels with BDI scores during early and late pregnancy and postpartum.

**Results:**

We found that vitamin D levels at 12–20 weeks were inversely associated with BDI scores both at 12—20 and at 34–36 weeks’ gestation (*P* < 0.05, both). For every one unit increase in vitamin D in early pregnancy, the average decrease in the mean BDI score was .14 units. Vitamin D levels were not associated with diagnoses of major depressive disorder or generalized anxiety disorder.

**Conclusions:**

In women at risk for depression, early pregnancy low vitamin D levels are associated with higher depressive symptom scores in early and late pregnancy. Future investigations should study whether vitamin D supplementation in early pregnancy may prevent perinatal depressive symptoms.

**Trial registration:**

https://clinicaltrials.gov/ Registration Number: NCT00711971

## Background

Antenatal depression may complicate up to 10–37 % of pregnancies, while as many as 27–54 % of pregnancies are complicated by antenatal anxiety [1–3]. Depression in pregnancy and postpartum is associated with significant morbidity [4, 5]. For example, perinatal depression has been associated with impaired mother-infant bonding and has also been associated with adverse outcomes of pregnancy, such as preterm birth, IUGR, and low birth weight [5–10]. In addition, depressive symptoms during pregnancy have been shown to be associated maternal morbidities such as increased risk for preeclampsia and operative deliveries [11].

It has been hypothesized that nutritional deficiencies may increase risk for depression [12]. For instance, a systematic review and meta-analysis of observational studies involving 31,424 non-pregnant adults found low vitamin D levels to be significantly associated with clinical diagnoses of depression [13]. Vitamin D is obtained by ingestion of vitamin D-containing foods or produced endogenously by the skin after exposure to ultraviolet B sunlight. Its relationship to depression was initially suggested based upon epidemiologic studies that noted an increase in depressive symptoms during winter months [14]. One hypothesized physiologic mechanism through which Vitamin D may act to affect depressive symptoms has emerged with the understanding that vitamin D acts as a neuroactive hormone, in addition to its role as a fat-soluble vitamin [15]. Supporting this concept, Eyles et al. demonstrated that vitamin D receptors are broadly distributed throughout the human brain [16] and animal studies have found that vitamin D deficiency or dietary manipulation/addition of vitamin D alters neurotransmitters that are known to be involved in depressive symptoms and depression [17–20]. Vitamin D may also play a role in neuroimmunomodulation and neuroplasticity, a proposed mechanism for the observed effect on mood [21].

There have been a number of studies in pregnant and postpartum women suggesting that this association exists in this population as well. For example, in a cohort of pregnant African-American women who were not selected based on predisposition to depression, Cassidy-Bushrow found early pregnancy low vitamin D levels to be associated with mid-pregnancy symptoms of depression [22]. In a follow-up study of a subset of this cohort, the authors also demonstrated an association of low early pregnancy vitamin D levels with postpartum depressive symptoms [23]. Similarly, Brandenbarg et al. found an association between low early pregnancy vitamin D levels and depressive symptoms measured at 16 weeks gestation [24]. The more recent Huang et al., study examined the association 25 OH vitamin D levels measured at 15 weeks gestation with depression and anxiety symptoms measured at that same time point [25].

Several studies have evaluated the association of vitamin D levels with postpartum symptoms of depression. Robinson, et al., in an unselected Australian cohort of Caucasian women, found vitamin D levels at 18 weeks’ gestation to be associated with postnatal depressive symptoms measured at 3 days postpartum [26]. Similarly, Gur, et al., found a significant associated between mid-pregnancy vitamin D levels and depression and depression screen scores measured longitudinally in the postpartum period among healthy women who were not considered at risk for depression [27]. In a longitudinal study carried out in the postnatal period, Murphy et al. found an association between vitamin D levels <32 ng/ml measured monthly for 7 months postpartum and depression symptom scores at these same time points [28]. By contrast, the recent Nielsen case-control Danish National Birth Cohort found that high levels of vitamin D in mid pregnancy were associated with antidepressant use in the postpartum period, an unexpected finding [29].

We conducted this study to evaluate whether plasma vitamin D levels, measured in early and late pregnancy were associated with depression symptom scores at 3 time points during pregnancy and at 6–8 weeks postpartum. This study differs from previously-reported investigations in that our subjects were selected based on predisposition to depression and both 25 [OH] vitamin D and depression scores were measured at several time points. We measured depressive symptom scores at several time points, using the Beck Depression Inventory to assess the severity of depressive symptoms over time [30–32]. We hypothesized that lower vitamin D levels during pregnancy would be associated with higher depression symptom scores during pregnancy and at 6–8 weeks postpartum.

## Methods

### Study design

This study is a secondary analysis of the data and blood samples of a cohort of women who had enrolled in the Mothers, Omega-3 & Mental Health Study trial, a prospective, double blind, placebo-controlled, randomized controlled trial designed to assess whether omega-3 fatty acid supplementation during pregnancy would prevent antenatal and postpartum depressive symptoms among pregnant women at risk for depression. The protocol for this study has been previously described [33]. The primary aim of this secondary analysis was to determine whether low vitamin D during pregnancy is associated with depressive symptoms as assessed by the Beck Depression Inventory score at three time points during pregnancy. As a secondary aim of this study, we evaluated whether vitamin D levels were associated with Mini International Neuropsychiatric Interview diagnoses of major depressive disorder (MDD), generalized anxiety disorder (GAD), or anxiety symptoms (MINI anxiety subtest, question 1a) [34].

### Study population and protocol

Between October 2008 and May 2011 we enrolled 126 pregnant women at risk for depression from prenatal clinics associated with The University of Michigan Hospital in Ann Arbor, Michigan, and St. Joseph Mercy Hospital in Ypsilanti, Michigan. We followed them prospectively throughout pregnancy (12–20 weeks [visit 1], 26–28 weeks [visit 2], and 34–36 weeks [visit 3]) and at 6–8 weeks postpartum [visit 5]. Women were invited to enroll based on risk factors for perinatal depression, including a past history of depression or postpartum depression, or an Edinburgh Postnatal Depression Scale (EPDS) score of 9–19. The EPDS, used to screen for depression risk in this study, has been accepted as a valid and reliable 10-item measure of perinatal mood [35]. Although a cut-off score of 11 is commonly used to screen for risk for major depression, EPDS cutoff scores as low as 9–10 have been shown to predict depression risk [3, 36–38]. We chose to use a cutoff value of 9 because we aimed to enroll women at risk for depression who however were not overtly depressed. Therefore, women with scores between a 9 and 19 (at risk for depression or mildly depressed) on the EPDS were eligible for randomization; women with scores > 19 were considered severely depressed and were ineligible for randomization.

Other inclusion criteria were: age ≥ 18 years, singleton gestation, and gestational age between 12–20 weeks. Additional exclusion criteria were: history of bleeding disorder, thrombophilia requiring anticoagulation, multiple gestation, bipolar disorder, current major depressive disorder, current substance abuse, lifetime substance dependence or schizophrenia. Women were also ineligible if they were taking omega-3 fatty acid supplements, antidepressant medications, or eating more than 2 fish meals per week [33]. All participating women were taking prenatal vitamins, which typically contain approximately 400 IU vitamin D [39]. Participants were not excluded from initiating antidepressant medications during the trial, if necessary [33].

Upon enrollment, participants were randomized to one of three groups: a) EPA-rich fish oil supplement (1060 mg EPA plus 274 mg DHA); b) DHA-rich fish oil supplement (900 mg DHA plus 180 mg EPA); and c) placebo. The aims and primary outcomes of the parent study are reported in separate publications [33, 40]. The primary outcome of the parent study, the Beck Depression Inventory score at 34–36 weeks gestation, and at 6–8 weeks postpartum, which was not influenced by either fish oil intervention at any time point in pregnancy, when compared with placebo [40].

### Psychological assessments or instruments

#### Beck depression inventory

We administered two psychological instruments, the Beck Depression Inventory and the Mini International Neuropsychiatric Interview, to enrolled study participants at enrollment (12–20 weeks), at 26–28 weeks, at 34–36 weeks, and at 6–8 weeks postpartum. Study participants self-completed the BDI. The BDI consists of 21 questions regarding depressive feelings (hopelessness and irritability), cognitions (guilt, feelings of being punished) and physical symptoms (fatigue, weight loss) and has been validated in the perinatal period. Higher total scores indicate more severe depressive symptoms. The BDI is ideal for repeat evaluation, as it is responsive to changes over time. We used the Beck Depression Inventory (BDI) to assess *depression symptom severity* [33, 40]. The test characteristics of the BDI in the perinatal period have been previously described [30, 31]. Normative values for BDI scores previously established in our population were used for the purpose of our sample size calculations [41].

Study staff with training in clinical psychology (masters or doctoral student level) administered the Mini International Neuropsychiatric Interview (MINI) in order to *diagnose* major depressive disorder (MDD) and Generalized Anxiety Disorder (GAD) [33, 40].

The MINI is a structured interview designed as a diagnostic tool for DSM-IV and ICD-10 psychiatric disorders. We used the MINI at enrollment (12–20 weeks), to identify ineligible participants. At subsequent visits (26–28 weeks, 34–36 weeks, and 6–8 weeks postpartum) we used the MINI to assess for development of major depressive disorder and to facilitate referral to appropriate mental health services. For our secondary outcomes we evaluated major depressive disorder, generalized anxiety disorder, as well as the generalized anxiety question on the MINI, “Have you worried excessively or been anxious about several things over the past 6 months?” We evaluated this anxiety question separately in order to assess anxiety symptoms that may be important but that did not meet criteria for generalized anxiety disorder.

#### Biomarkers

Maternal venous blood was drawn at enrollment at 12–20 weeks (*n* = 117) and at 34–36 weeks gestation (*n* = 112). The blood was drawn after a 4 h fast. All blood samples were processed within 12 h and aliquots of serum or plasma were frozen at −70° Celsius under argon until analysis.

Blood sample analyses from the parent study included the omega-3 fatty acids eicosapentaenoic acid (EPA) and docosahexaenoic acid (DHA) from serum. These analyses have been previously described [33, 40]. For this secondary analysis, we measured serum 25-hydroxyvitamin D (25-OH-D) in stored plasma aliquots. Although 1, 25-Dihydroxyvitamin D (not 25-OH-D) is the biologically active form of vitamin D, circulating levels of 1, 25-Dihydroxyvitamin D are not a good representation of tissue levels, and tissue levels are not easily measured. On the other hand, 25-OH-D is the major form of circulating vitamin D and is highly stable for analysis from stored serum or plasma. Thus, most of the current literature uses 25-OH-D as the assessment of overall vitamin D status [28, 42, 43]. This measurement was performed using a 25-OH-D 125I Radioimmunoassay kit from DiaSorin, Stillwater, Minnesota, U.S.A [44]. Inter-assay variations have been shown to be <10 % at levels of 25-OH-D between 15–67 ng/ml.

### Statistical analysis

We evaluated mean vitamin D levels and standard deviations at 12–20 weeks and at 34–36 weeks and used Pearson’s correlation to evaluate the relationship between time points. We used the *T*-Test procedure to evaluate the relationship between vitamin D 1 at enrollment and seasonality, with “winter” being defined as “December through February”.

In our primary analysis, we assessed the relationship between vitamin D as a continuous variable and BDI scores at 12–20 weeks, 34–36 weeks and the 6–8 week postpartum visits. Using a generalized linear models (ANCOVA) approach we entered winter and vitamin D at visit 1 into the model as predictors of the BDI score at visit 1 (study entry), at 34–36 weeks gestation and at 6–8 weeks postpartum.

We used repeated measures ANOVA to fit the vitamin D level at baseline and in late pregnancy to the square root of the BDI score at visits 1, 2, 3, and 5, adjusting for “winter”, baseline BDI, baseline vitamin D and most recent vitamin D. We transformed the BDI by its square root to correct for skewed (non-normal) distribution. Evaluation of psychiatric diagnoses by the MINI diagnostic tool was analyzed using Fisher’s Exact test.

To compare outcomes according to vitamin D sufficiency versus insufficiency in our population, we performed a secondary analysis selecting a vitamin D level of ≥ 20 ng/mL at enrollment (*n* = 98) as our reference group and <20 ng/mL (*n* = 19) as our “low vitamin group. We defined these two groups based upon assessment from the Institute of Medicine that most individuals are assured good bone health with levels that are 20 ng/ml or higher, as well as the Endocrine Society’s Clinical Practice Guideline definition of vitamin D deficiency of 25 OH vitamin D levels <20 [43, 45]. No threshold has been established for prevention of other diseases hypothesized to be related to vitamin D status [43, 45–47]. Of note, the Endocrine Society has suggested a vitamin D target for pregnant women of ≥30 ng/ml [43].

We used stepwise linear regression analyses to better define the relationship between “low vitamin D” and BDI score while adjusting for potentially confounding effects. Variables included in these analyses were those relating to the parent study (serum DHA and EPA levels, which have been hypothesized to be associated with depressive symptoms [33, 40] along with other factors thought to be potentially associated with depressive symptoms (age, tobacco use, obesity [defined as BMI ≥30], initiation of anti-depressant medications). In this model, low vitamin D and other variables were considered categorically (DHA <2.0 as % of total fatty acids, EPA <0.1 as % of total fatty acids, age >35, positive tobacco use, BMI >30, and positive initiation of anti-depressant medications). Because there are no published normative values for DHA and EPA in pregnancy, we chose to define low DHA as <2 (approximately 1 standard deviation below the mean in our sample), and low EPA as <0.1 (also approximately 1 standard deviation below the mean in our sample). We evaluated the association of vitamin D levels at visits 1 and 3 with “winter”, using Fisher’s exact test.

We used descriptive statistics to compare characteristics of the reference group and the “low vitamin D” group. Student’s test was used for continuous variables that were normally distributed, the Mann-Whitney test was used for non-parametric comparison of ordinal variables, and Fisher’s Exact test was used for rare categorical variables.

All statistical analyses were performed using SAS Version 9.3 (SAS Institute, Inc., Cary, NC).

## Results

**Fig. 1:**
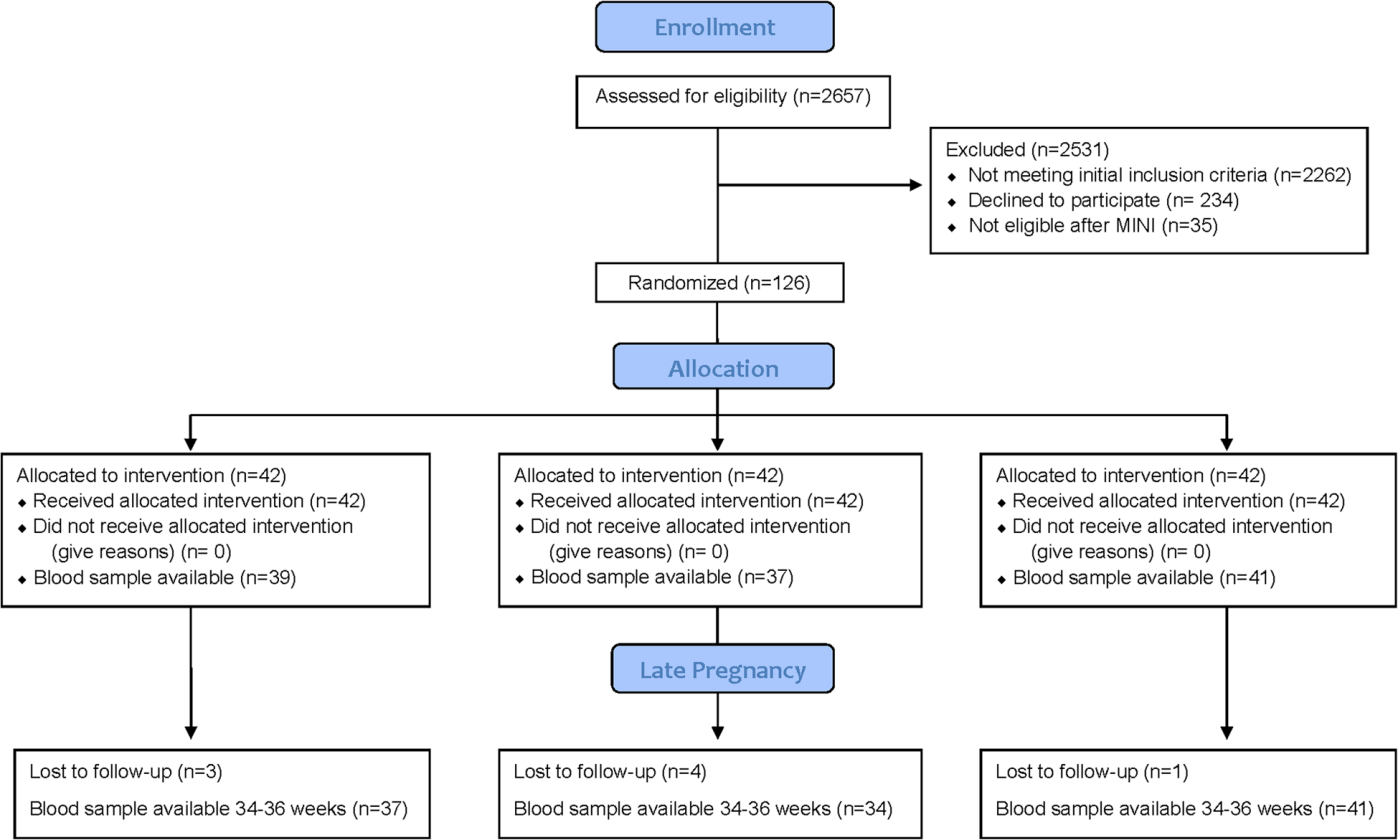
Consort diagram

In the parent study, 126 women were enrolled and were randomly assigned to receive EPA-rich fish oil, DHA-rich fish oil, or placebo. Of these, 8 women discontinued trial participation entirely and were lost to follow up. Plasma samples were available for 117 participants at 12–20 weeks and 112 participants at 34–36 weeks. Complete study datasets (including all variables of interest) were available for 105 participants. The flow of study participants and blood samples is shown in Fig. 1. The demographic characteristics of the cohort have been previously described [40]. In brief, the study population was 81.3% white, with African-Americans representing 8.5 % of the cohort. The remainder of the study cohort were of Asian, Native American, Hispanic, or Pacific Islander ethnicity. The ethnic and racial characteristics did not differ significantly among the randomized groups [40]. Because of the small proportion of non-white participants, with 8 % of the total cohort for the purpose of the analyses, the non-white participants were combined. In addition, our subjects were relatively homogeneous in socioeconomic status, with at 89 % of our study population having at least some college education.

Participants characteristics were stratified by possible vitamin D deficiency (25 (OH) D <20 ng/mL) for descriptive purposes (Table 1). The mean vitamin D level for the aggregate study population was 28.17 ng/ml+/− 8.25 and 3.1.84 ng/ml +/− 10.63at 12–20 weeks and 34–36 weeks respectively. Vitamin D levels at 12–20 weeks were strongly correlated with vitamin D levels at 34–36 weeks (Pearson’s *ρ* = 0.60, *P* < .0001). Vitamin D levels drawn in winter were significantly lower than vitamin D levels drawn during other seasons (mean vitamin D =29.0 +/− 8.2 versus 26.5 +/− 7.6, *P* < 0.05).

**Table 1:** Baseline Characteristics Stratified by Vitamin D level at 12–20 weeks

Parameter	Vitamin D ≥20 ng/mL	Vitamin D <20 ng/mL	*P* – value
	(*N* = 98)	(*N* = 19)	
Age			^a^0.03^b^
Mean, S.D.	30.8, 5.04	27.8, 5.04	
Gravidity			0.17^c^
Mean, S.D.	2.36, 1.49	3.00, 1.91	
Parity			0.27^c^
Mean, S.D.	.89, 0.98	1.15, 1.12	
Gestational age (weeks) at enrollment			0.57^b^
Mean, S.D.	16.3, 2.41	16.7, 2.60	
White N (percent)	82 (84)	14 (74)	0.38^d^
Non-White N (percent)	16 (16)	5 (26)	
Self-reported history of depression			1.00^d^
N (percent)	79 (81)	15 (79)	
Reported tobacco use			^a^0.004^d^
N (percent)	6 (6)	6 (32)	
BMI			0.10^c^
Mean, S.D.	27.62, 6.82	31.00, 7.85	
October-March at enrollment			0.81^d^
N (percent)	55 (56)	10 (53)	
EPA (as % of total fatty acids)			0.11^c^
Mean, S.D	.37, .21	.32, .24	
DHA (as % of total fatty acids)			0.44^c^
Mean, S.D.	3.18, 1.32	2.91, 0.80	
BDI at baseline	7.4, 4.9	10.2, 7.1	0.11

*S.D*. standard deviation, *BMI* body-mass index, ^a^ = statistically significant

^b^Student’s *t*-test

^c^Mann-Whitney test

^d^Fisher’s Exact test

As previously discussed, we selected a vitamin D level ≥ 20 ng/mL (*N* = 98) for our reference group and <20 ng/mL (*N* = 19) for our “low vitamin D” group for sub-analysis. The “low vitamin D” group was younger (mean age 27.8 years versus 30.8 years, *P* 0.03) and more likely to report tobacco use (32 % versus 6 %, *P* 0.004). The remaining descriptive characteristics of interest were not significantly different between the two groups (Table 1). The frequency of vitamin D <20 ng/ml at 12–20 versus 34–36 weeks was 16 % and 12 % of our total population. Mean values and standard deviations for the two groups are shown in Table 2.

**Table 2:** Vitamin D Characteristics

Parameter	Vitamin D ≥20 ng/mL	Vitamin D <20 ng/mL
12–20 weeks		
n (percent)	98 (84)	19 (16)
Mean, S.D.	30.57, 6.65	15.78, 2.58
34–36 weeks		
n (percent)	98 (88)	14 (12)
Mean, S.D.	34.36, 8.69	14.21, 4.37

*S.D*. standard deviation

For our primary outcome, we evaluated the relationship between vitamin D levels at two time points and BDI scores at 12–20 weeks, 34–36 weeks and 6–8 weeks postpartum. Using analysis of covariance (ANCOVA), and adjusting for season, (winter/non-winter), we found that vitamin D at 12–20 weeks was a significant predictor of the BDI score at 12–20 (*P* < 0.05) weeks and at 34–36 weeks gestation (*P* < 0.05). For every one unit increase in vitamin D in early pregnancy there was an approximate 0.14 unit decrease in the BDI score at visit 1 (95 % confidence interval −0.26, −0.017) and visit 3 (−0.27, −0.011). Vitamin D at visit 34–36 weeks did not significantly predict the BDI score at that same time point.

We next performed repeated measures analysis, evaluating for association of the most recent vitamin D value with the BDI score adjusting for winter, baseline BDI score, most recent BDI score, most recent DHA level and group assignment. The model was fit to the square root of the BDI score due to skewed distribution. There was a non-significant trend towards association of the most recent vitamin D level with the BDI score (*P* = 0.07). However, only the baseline BDI score was significantly associated with the BDI score on repeated measurements (*P* < 0.001).

When evaluating “low vitamin D” as a categorical variable, there was no association seen between vitamin D level at 12–20 weeks and BDI score at that time (*P* = 0.11) or with postpartum BDI score (*P* = 0.97). However, “low vitamin D” at enrollment was significantly associated with higher BDI score at 34–36 weeks gestation (*P* = 0.05).

Because our study participants were drawn from a parent study looking at omega-3 fatty acids (DHA and EPA) and their relationship to depression, and to adjust for other potential confounders, we conducted a stepwise linear regression to better understand the association of vitamin D with the BDI score. In this adjusted model, “low vitamin D”, at visit 1 (again, as a categorical variable) was associated with the BDI at 34–36 weeks’ gestation (*P* = 0.01), while there was a trend toward association of low DHA (*P* = 0.06) and low EPA (*P* = 0.09) with higher BDI scores at that time point as well (R^2^ for the model = 0.12). There was no association between low vitamin D and postpartum BDI scores in the regression analysis. The remaining variables we evaluated (age, tobacco use, obesity (BMI) and initiation of anti-depressant medications) did not significantly impact this model. That is, age, tobacco use, BMI, and initiation of antidepressant medications were not significantly associated with the BDI scores. We found that 12 % of the variance in the BDI score at 34–36 weeks was predicted by low vitamin D levels, low EPA, and low DHA at 12–20 weeks.

Vitamin D levels were not associated with a higher risk of major depressive disorder, generalized anxiety disorder, or a positive response to anxiety question 1a at any time. Of interest, 38.5 % of our study population reported a positive response to anxiety question 1a at 34–36 weeks, perhaps identifying this as a time of particular vulnerability to such symptoms.

## Discussion

### Main findings

This study presents evidence that low vitamin D levels in early pregnancy (12–20 weeks) are significantly associated with higher depression symptom scores at in early and late pregnancy in a group of women at risk for depression. Conversely, we found that although there was a trend toward an association of most recent vitamin D levels with the BDI score in later pregnancy and postpartum, this trend was not significant and largely disappeared after adjustment for the baseline BDI score. Low vitamin D levels (at any time point) were not significantly associated with higher postpartum depression symptom scores.

## Strengths and limitations

Our study had four main strengths, one of which was its prospective, longitudinal study design, allowed for measurement of depressive symptoms at several time points during pregnancy, as well as at 6 weeks postpartum. Another strength of this study is that we were able to measure vitamin D longitudinally as well, both in early and late pregnancy. A third strength of this study was that we assessed both depression symptom scores (using the BDI) as well as depression diagnoses (using the MINI). Lastly an area of strength in this study was that it evaluated women at risk for depression, allowing for our outcome of interest (depressive symptoms) to be moderately prevalent in our study cohort. The lack of association observed between vitamin D level in early or late pregnancy and the 6–8 week postpartum BDI score may be explained by the complexity of the pathogenesis of postpartum depression symptoms, or it may represent a true negative finding.

Our study had four main limitations. This was a secondary analysis of a randomized, controlled trial, designed to detect a reduction in BDI score following intervention with omega-3 fatty acids. As such, results must be interpreted with caution. Specifically, although we attempted to control for confounding related to omega-3 fatty acid levels, it is still possible that the intervention, or even the participation in a clinical trial itself, led to inappropriate conclusions. An additional limitation of this study is that the sample size for the randomized controlled trial was chosen based on the hypothesized response to the omega-3 fatty acid interventions under study on the BDI score, rather than as a study to detect an association. Another potential limitation of the study is that we used the BDI rather than the EPDS to assess depressive symptom severity. We specifically chose the BDI for this assessment in the parent trial because the expected BDI scores in our population had been previously established [41]. An additional limitation of our study was that we were unable to explore the potentially modulatory effects of inflammatory cytokines on the relationship between vitamin D and depressive symptoms [23].

Our study found an association between low vitamin D levels in early pregnancy and depressive symptoms during pregnancy but not postpartum. In the published literature, both low and high vitamin D levels have been associated with perinatal depression, a finding that others have hypothesized may be due to a 24-hydroxylase-based degradation mechanism responding to abnormally high concentrations of vitamin D [29].

The main finding of our study is that there exists a relationship between low early-pregnancy vitamin D levels and depression symptoms in early and late pregnancy in women at risk for depression. Our regression model suggested that 12 % of the variance in BDI score at 34–36 weeks was predicted by a combination of low vitamin D, low EPA and low DHA levels in early pregnancy. Although this may not appear to be a strong relationship, for complex psychological and psychiatric diagnoses, this magnitude of association may indicate a relationship worthy of further investigation. Vitamin D supplementation is a low cost and safe intervention in pregnancy [48]. If randomized studies show a benefit for vitamin D for prevention or treatment of perinatal depression, supplementation may benefit maternal fetal and neonatal health [49, 50].

## Conclusions

Our findings suggest that lower vitamin D levels in early pregnancy are associated with depressive symptoms in early and late pregnancy. This association may be attributed to a true effect of nutritional deficiency or alternately might be related to differences in vitamin D metabolism between women who are predisposed to depression and those who are not. Important future areas of research would include randomized controlled trials of vitamin D supplementation for women who are screen for depression risk in early pregnancy and who are found to have low vitamin D levels. Such future research should also explore any potential interaction between inflammatory cytokines and vitamin D on depression symptoms.

## Abbreviations

BDI, Beck Depression Inventory; EPDS, Edinburgh Postnatal Depression Scale; Visit 1, 12–20 weeks gestation, study enrollment; Visit 3, 34–36 weeks gestation; Visit 5, 6–8 weeks postpartum
